# Enhanced oil recovery from sandstone reservoir using cationic hyperbranched polyamidoamine dendrimer surfactant flooding under real reservoir conditions

**DOI:** 10.1038/s41598-025-17757-w

**Published:** 2025-09-12

**Authors:** Samah A. M. Abou-alfitooh, Ammona S. Mohamed, Fatma M. Abdelhafiz, Ahmed Zahran, R. Hosny

**Affiliations:** 1 EOR Lab, Production Department, Egyptian Petroleum Research Institute, 1 Ahmed El Zomor St., Nasr City, Cairo 11727 Egypt; 2 Core Lab Center, Egyptian Petroleum Research Institute (EPRI), 1 Ahmed El Zomor St., Nasr City, Cairo 11727 Egypt; 3 Petrochemicals Department, Egyptian Petroleum Research Institute (EPRI), 1 Ahmed El Zomor St., Nasr City, Cairo 11727 Egypt; 4 PVT Lab, Production Department, Egyptian Petroleum Research Institute (EPRI), 1 Ahmed El Zomor St., Nasr City, Cairo 11727 Egypt

**Keywords:** Enhanced oil recovery, PAMAM dendrimer surfactant, Sandstone reservoirs, Interfacial tension, Wettability alteration, Chemistry, Engineering

## Abstract

This study investigates the application of a synthesized cationic hyperbranched polyamidoamine (PAMAM) dendrimer surfactant (G2-C12) as a novel chemical agent for enhanced oil recovery (EOR) in sandstone reservoirs under realistic high-salinity and high-temperature conditions. The dendrimer was synthesized via a divergent approach involving Michael addition, amidation, and quaternization, yielding a structurally defined amphiphilic macromolecule. Its performance was evaluated through interfacial tension (IFT) measurements, salinity-dependent behavior, wettability alteration tests, and core flooding experiments. The surfactant reduced IFT from 27 mN/m to 5 mN/m at 2000 ppm, which was identified as the critical micelle concentration (CMC). Contact angle measurements confirmed a significant shift from oil-wet to strongly water-wet conditions (10.87°–34.57°). Additional salinity tests established the optimal brine strength, while core flooding results demonstrated an incremental oil recovery of 29.09% at reservoir-representative conditions. These findings underscore the dendrimer’s dual-function mechanism, operational stability, and strong potential as a high-performance EOR surfactant under challenging field environments.

## Introduction

The increasing global energy demand necessitates innovative strategies to maximize crude oil extraction from existing reservoirs^1^. While primary and secondary recovery methods recover a significant portion of the original oil in place, a substantial amount remains trapped within the porous structure of reservoir rocks due to capillary forces and unfavorable wettability. Enhanced Oil Recovery (EOR) has thus emerged as a critical technique to supplement these conventional methods^2,3^. Among various EOR approaches, chemical EOR, particularly surfactant flooding, stands out due to its unique dual capacity to mobilize this residual oil^4–6^.​.

The effectiveness of surfactant flooding hinges on two primary mechanisms: Interfacial Tension (IFT) Reduction and Wettability Alteration. Surfactants, being amphiphilic molecules, dramatically lower the IFT between oil and water, reducing capillary forces and allowing floodwater to more easily displace and mobilize residual oil. Simultaneously, surfactants can modify the rock’s surface properties, changing its preference from oil-wet to more water-wet or mixed-wet conditions. This alteration allows water to spread more easily on the rock, detaching oil from pore walls and facilitating its flow, thereby improving both microscopic displacement and macroscopic sweep efficiencies^7,8^.

Surfactants are broadly categorized based on their head-group charge, each with distinct advantages and limitations in EOR. Anionic surfactants are widely studied for their strong IFT reduction capabilities and are generally effective in low to moderate salinity. However, they can precipitate or adsorb significantly in high-salinity brines or on positively charged rock surfaces. Cationic surfactants, with their positive charge, excel at altering wettability, particularly in carbonate or mixed-wet reservoirs, but may not achieve ultra-low IFT as readily as some anionic types and can suffer from high adsorption on negatively charged sandstone^9^. Nonionic surfactants, lacking a charge, exhibit less sensitivity to salinity and temperature but typically do not achieve the ultra-low IFT required for significant microscopic displacement, often serving as co-surfactants^10^. Amphoteric/Zwitterionic surfactants, possessing both charges, offer flexibility across pH ranges, reduced salinity sensitivity, and improved IFT reduction and wettability alteration, often with lower adsorption^11^.

Despite their potential, conventional surfactants often face significant limitations under harsh reservoir conditions, such as high salinity, elevated temperatures, and rock adsorption losses, often failing to deliver consistent field-like results^12^. For example, surfactant solutions that perform well under laboratory conditions often fail to deliver consistent results when exposed to high-salinity brines and reservoir temperatures^13^.

Several efforts have been made to improve the effectiveness of surfactants under field-like conditions. Rezk and Allam demonstrated that core flooding using 2 g/L sodium lauryl sulfate increased oil recovery by about 16% following water flooding, primarily due to a significant reduction in IFT^14^. Wu et al. also reported an additional oil recovery of 4.43% using sodium dodecyl sulfate after waterflooding^15^. In parallel, plant-based surfactants and hybrid systems have shown promise in modifying rock wettability wet^16,17^. Moreover, mixed surfactant systems have been proven more effective than individual surfactants, achieving over 10% improvement in recovery under harsh conditions^18^. These findings underscore the critical role of both IFT reduction and wettability alteration in enhancing oil displacement^19^.

To overcome the challenges associated with conventional surfactants, dendrimer-based molecules have emerged as a promising alternative in enhanced oil recovery (EOR)^20^. Dendrimers are nanoscale, highly branched polymers distinguished by their well-defined architecture, high density of surface functional groups, and outstanding chemical and thermal stability^21^. Among them, cationic polyamidoamine (PAMAM) dendrimers exhibit amphiphilic characteristics and structural uniformity, enabling them to efficiently reduce interfacial tension (IFT) and alter rock wettability, even under high-salinity conditions^22^. Recent investigations have demonstrated that dendrimer surfactants can maintain interfacial activity and wettability modification efficiency where conventional anionic or nonionic surfactants fail, particularly at salinities exceeding 100,000 ppm and temperatures above 70 °C. This stability is attributed to their branched molecular framework, tunable terminal groups, and strong electrostatic interactions with mineral surfaces. Despite these advantages, their application in EOR remains limited, with few studies addressing their behavior under true reservoir-like conditions^23^. This study addresses this gap by synthesizing a novel cationic hyperbranched PAMAM dendrimer surfactant (G2-C12) and evaluating its interfacial, wettability, and oil recovery performance under realistic high-temperature, high-salinity sandstone reservoir conditions.

This work is among the few to test a structurally defined dendrimeric surfactant under true reservoir-like conditions, using actual sandstone cores at elevated salinity and temperature, and thus aims to bridge this gap by synthesizing a novel cationic hyperbranched PAMAM dendrimer surfactant (G2-C12) and evaluating its potential for enhanced oil recovery (EOR). The research investigates the physicochemical performance of the dendrimer in terms of interfacial tension reduction and wettability alteration, and validates its oil recovery efficiency through core flooding experiments. By addressing the limitations of conventional surfactants, this study offers a viable and innovative pathway to improve EOR processes in complex and challenging reservoir environments.

## Materials and experiments

### Materials

The chemicals utilized in this study included piperazine (99%, Acros Organics), ethylene diamine (99%, Acros Organics), methyl methacrylate (99%, Acros Organics), manganous chloride (98%, Acros Organics), and alkyl bromides—namely butyl bromide (99%, Acros Organics), octyl bromide (99%, Acros Organics), and dodecyl bromide (99%, Acros Organics). Acetone (99.5%, ADWIC) was used as a solvent. High-purity distilled water was employed throughout the experiments, along with calcium chloride (99.9%, Merck) and sodium chloride (99.9%, Merck) for brine preparation. Crude oil with an API gravity of 29 was used as the hydrocarbon phase, while subsurface sandstone core plugs served as the porous medium in core flooding experiments. Toluene (99.89%, Merck) and methanol (99.89%, Merck) were used as cleaning agents and in phase behavior studies. The crude oil was sourced from the Gulf of Suez Basin, Egypt. These core plugs were obtained from the Bahariya Formation in the Western Desert, Egypt. These salts were commercially available laboratory-grade chemicals sourced from local suppliers in Egypt.

### Core materials

A total of 3 core plugs (A, B & C) of 1.5-inch diameter have been drilled using a diamond core drill with tap water as a bit of coolant and lubricant. The obtained cylindrical core plugs were trimmed with a diamond core saw to form a uniform right cylinder. Hydrocarbons were extracted from the plug samples in a hot solvent reflux soxhlet using toluene. Any salt present is leached from the samples using methyl alcohol in a solvent reflux soxhlet extractor. The samples were dried in a regular oven at 100 °C. The cores were weighted, where the bulk volume (B_V_) was calculated using a digital Vernier caliper, and then the grain volume (GV) was calculated through a helium porosimeter instrument, hence the pore volume (PV) and the porosity (Ø) were calculated.

### Preparation of surfactant

#### Synthesis of hyperbranched polyamidoamine (PAMAM) dendrimer

**Fig. 1:**
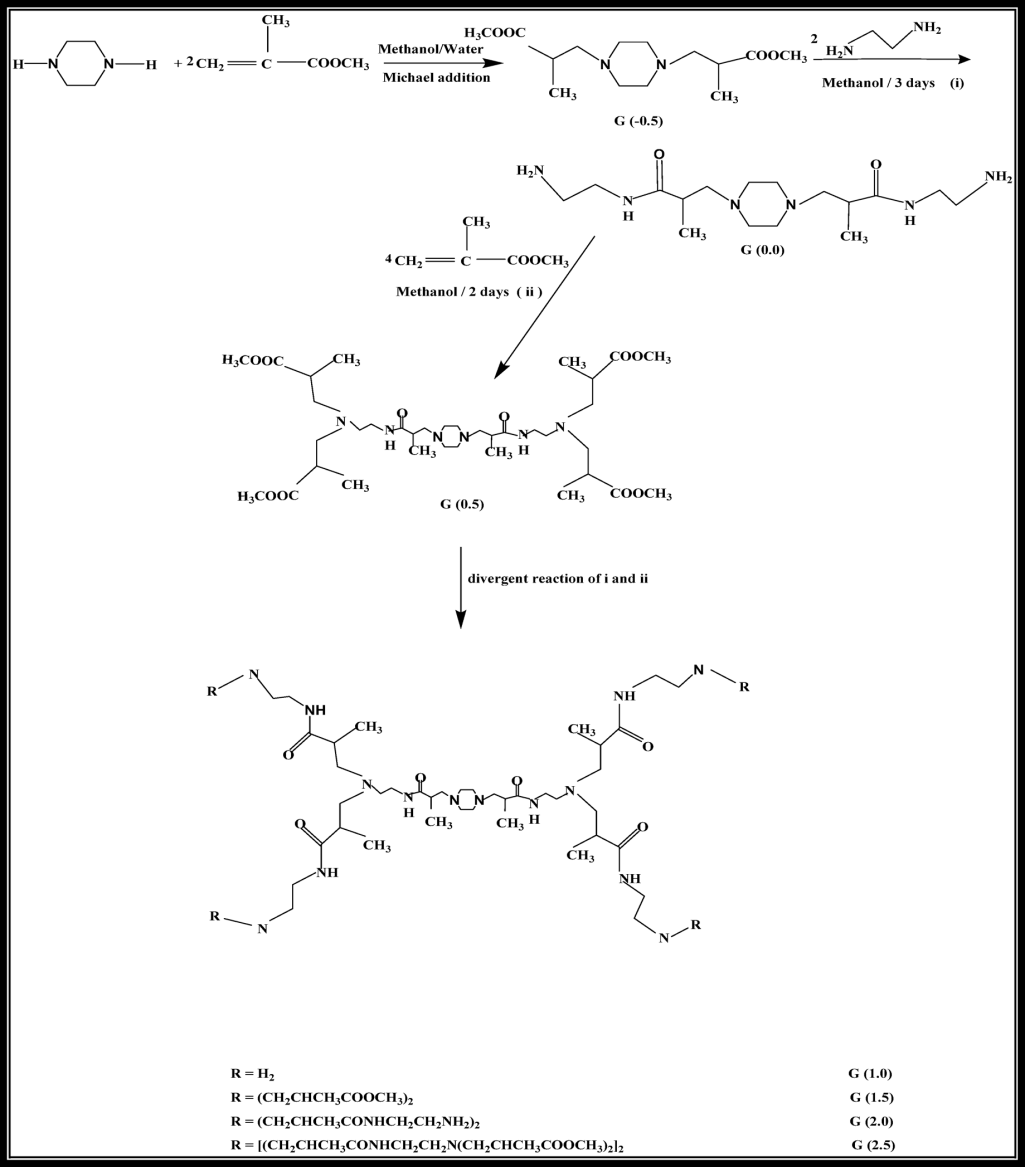
Schematic illustration of the divergent synthetic pathway for PAMAM dendrimer^26^.

The synthesis of a cationic hyperbranched PAMAM dendrimer, based on a piperazine core, followed a divergent approach involving Michael addition and amidation reactions. The Michael addition created intermediate generations (–0.5, 0.5, 1.5, 2.5), while the amidation step yielded full-generation amine-terminated PAMAM dendrimers (0, 1, 2), as depicted in Fig. 1^24^.

#### Michael addition reaction for G (–0.5, 0.5,1.5, 2.5) PAMAM dendrimer

In this step, 20 mmol piperazine was reacted with 40 mmol methyl methacrylate in the presence of 0.4 g MnCl_2_ as a catalyst, using a methanol-water solvent mixture (30 mL, 50:50). The reaction was carried out at room temperature with stirring for 20 min. After completion, the resulting brown solution was filtered to remove MnCl_2_, and methanol was evaporated under reduced pressure to obtain G-0.5 as a yellow, viscous product with an 85% yield Fig. 1^25,26^.

For G (0.5, 1.5, 2.5), the process was slightly modified. Methyl methacrylate (4.069 mol) was dissolved in 200 mL methanol and cooled in an ice bath. A solution of G-0.0 hyperbranched PAMAM (0.833 mol) in 200 mL methanol was added drop wise under continuous stirring for 2 h. The mixture was then allowed to reach room temperature (approximately 25 °C) and stirred for 48 h. Excess methyl methacrylate and the solvent were removed using a rotary evaporator at 45 °C, resulting in a methyl ester-terminated PAMAM dendrimer (G-0.5, 1.5, 2.5).

#### G (0.0, 1.0, 2.0) PAMAM dendrimer amidation reaction

**Fig. 2:**
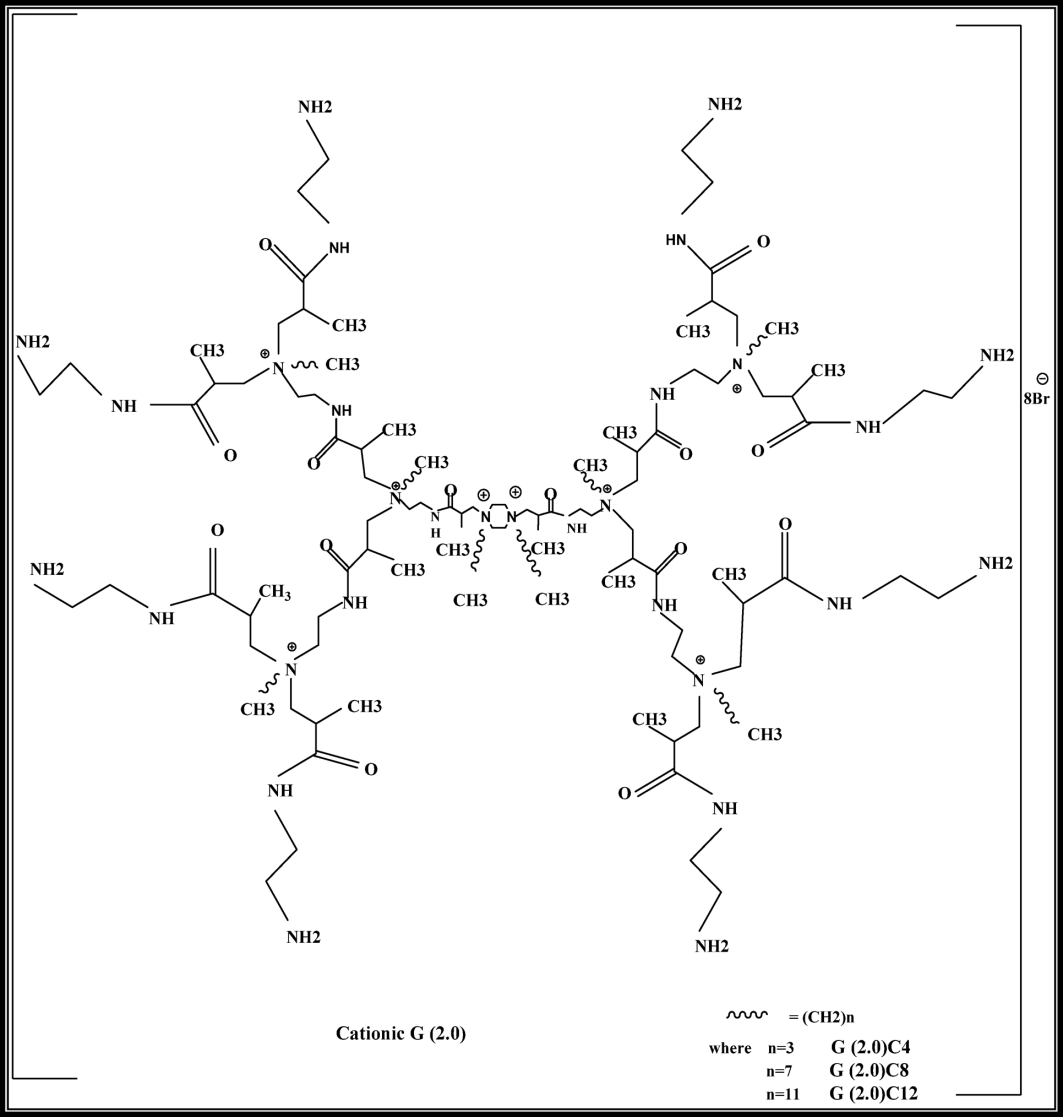
Synthesis scheme of cationic PAMAM dendrimer G (2.0)^26^.

For the amidation step, a solution of 1 mol G-0.5 PAMAM in 100 mL methanol was added dropwise to a stirred solution of 2 mol ethylene diamine in 300 mL methanol at 0 °C. The addition rate was controlled to maintain the temperature below room temperature. After the addition, the reaction mixture was stirred for 72 h at room temperature. The solvent and unreacted ethylene diamine were removed under reduced pressure at a temperature below 50 °C, yielding G-0.0 PAMAM as a deep yellow, viscous product Fig. 2. Subsequent syntheses followed the same procedure, producing generations G-1.0 and G-2.0^27^.

#### Quaternization of PAMAM dendrimer

To quaternize the dendrimer, generation 2 PAMAM was refluxed with dodecyl bromide in a methanol-acetone mixture at 70–80 °C for 24 h. The solvent mixture was then evaporated, and the product was dissolved in methanol and extracted with hexane to remove unreacted alkyl halide. The final product, G2-C12, was obtained in its pure form^28^.

### Characterizations of the prepared surfactant

The synthesized cationic PAMAM dendrimer (G2-C12) was characterized to confirm the success of the quaternization process and to validate its structural integrity. Fourier-transform infrared spectroscopy (FTIR) analysis revealed characteristic absorption bands corresponding to amide (–CONH–) linkages and quaternary ammonium groups, indicating the completion of both amidation and quaternization reactions. Proton nuclear magnetic resonance (^1^H NMR) spectroscopy confirmed the presence of methylene protons along the dendritic backbone and the terminal dodecyl chains, further verifying the structural modification. Carbon-^13^ NMR (Nuclear Magnetic Resonance) analysis appeared Peaks corresponding to amide (–C(= O)–NH–), and other functional groups can be identified, providing information about the functionalization of the dendrimer. Additionally, Elemental analysis (CHN) supported the expected elemental composition of the final product, with an observed increase in carbon content reflecting the incorporation of the long alkyl chains. All these analyses confirmed the chemical structure of cationic PAMAM dendrimer (G2-C12)^26^.

### Preparing synthetic formation water

A synthetic formation water of a total salinity of 10%wt. (100,000 ppm) was used in this study, where a ratio of 20:80 was adjusted for calcium chloride, and sodium chloride, respectively.

### Test techniques of characterization

Some tests were performed to study and analyze the effect of the new surfactant in EOR as follows:

#### Interfacial tension studies

Interfacial tension is the force that acts at the interface between two immiscible liquids. This force is caused by the imbalance of intermolecular forces at the interface^29,30^. The interfacial tension was measured using a theta optical tensiometer at 25 °C. Solutions of varying concentrations (0.0, 0.1, 0.15, and 0.2% w/v) of the dendrimer surfactant were tested. We used synthetic formation water (with a salinity of 100,000 ppm) to prepare various surfactant solutions simulating reservoir conditions.

#### Contact angle

Wettability alterations were assessed using the sessile drop method, utilizing the Biolin Scientific Company’s theta optical tensiometer from Finland^31^. Rock slices were analyzed for contact angles with crude oil, formation water, and surfactant solutions^32^. For this purpose, a number of thin rock slices of interest were cut using a trimming machine, and then an end face grinder was utilized to make the rock surfaces leveled. The rock slices were used without cleaning from crude oil to represent the reservoir. Three droplets of the crude oil, formation water, and the surfactant slug of size 8 µL for each were placed on the rock sample at different rock surface locations using a specific syringe. Rock tendency to a certain fluid (wettability) depends on the fluid/air/rock contact angle formed on the rock surface. For the liquid of interest, a series of enlarged photographs was taken over time using a digital camera. Measurements were conducted until equilibrium was reached. At room temperature, the images were analyzed using Attention Theta Software (Biolin Scientific, Version 4.1.0).

### Fluid characterization

The kinematic viscosities of fluids, including crude oil, and formation water, were measured in centistokes (cSt) at a reservoir temperature of 70 °C. This was achieved using an Ostwald viscometer, a U-shaped glass tube, placed in a water bath to maintain the desired temperature. The dynamic viscosity, expressed in centipoise (cP), was calculated by multiplying the kinematic viscosity by each fluid’s specific gravity (SG). Additionally, the densities of these fluids were also measured at the reservoir temperature using a pycnometer, which was similarly immersed in a water bath for temperature control. Finally, The API gravity was calculated through the specific gravity of the crude oil which is the density of the crude oil relative to the density of water at the same temperature (ambient or reservoir temperature).

### Core flooding test

The core samples were vacuumed and then saturated with brine containing 100,000 ppm salinity at a pressure of 3000 psi. They were left to saturate for one day to ensure complete absorption. After saturation, the cores were weighed again to confirm they were 100% saturated with brine. A stainless-steel core holder was used to hold the fully saturated core samples, where a temperature of 70 °C and a confining pressure of 4000 psi were applied. The original oil in place (OOIP) and initial water saturation (S_wi_) were evaluated through crude oil flooding of a fully brine-saturated core sample. Secondary flooding with formation water was conducted to assess the residual oil saturation until no more oil was displaced from the tested sample. Various surfactant concentrations (1 х 10^3^, 1.5 х 10^3^, and 2 х 10^3^ ppm) were applied to determine the optimal dosage for maximum oil recovery.

## Results and discussion

### Interfacial tension measurements

Interfacial tension (IFT) between crude oil and the injected aqueous phase is a critical factor in chemical enhanced oil recovery (EOR), as it governs the capillary forces that trap residual oil within porous media. A substantial reduction in IFT lowers the capillary pressure barrier, facilitating the mobilization of trapped oil, in accordance with the Young–Laplace equation (Pc = 2γcosθ/r), where γ is the IFT, θ is the contact angle, and r is the pore throat radius.

Table 1 presents the IFT values measured at different concentrations of the cationic PAMAM dendrimer G2-C12. In the absence of surfactant (0 ppm), the IFT was 30 mN/m with distilled water and 27 mN/m with formation water, indicating high capillary resistance. Upon adding the dendrimer surfactant, IFT decreased sharply, reaching 5 mN/m at 2000 ppm, demonstrating the surfactant’s strong interfacial activity.

This significant reduction is attributed to the amphiphilic nature of the dendrimer: its hydrophobic interior interacts with the oil phase, while the cationic hydrophilic terminals orient toward the aqueous phase, forming a stable interfacial layer. The branched structure and high surface functionality of PAMAM likely promote dense interfacial packing, which disrupts cohesive forces between the oil and water phases.

To determine the critical micelle concentration (CMC), additional IFT measurements were performed at 2500 ppm and 3000 ppm. The results showed negligible change beyond 2000 ppm, confirming that IFT had plateaued. This behavior indicates that the CMC of the G2 C12 surfactant lies around 2500 ppm. Identifying this concentration is important, as surfactant performance typically stabilizes once micellization occurs, beyond which further IFT reduction becomes minimal.

Each IFT measurement was performed in triplicate to ensure accuracy and reproducibility. The results presented in Table 1 are average values, and the corresponding standard deviations are included to reflect the consistency of the measurements. The low standard deviation values observed confirms the stability of the dendrimer surfactant and the reliability of the experimental procedure under the tested conditions.

The observed decrease in IFT from 27 mN/m to 5 mN/m at 2000 ppm is notably significant, especially under high-salinity conditions (10 wt% NaCl/CaCl_2_ brine). Compared to previous studies, this level of IFT reduction demonstrates a superior performance. For instance, Rezk and Allam reported an IFT reduction to approximately 12 mN/m using sodium lauryl sulfate at 2 g/L under similar salinity conditions^33^, while Wu et al. observed an IFT of 14 mN/m using sodium dodecyl sulfate^15^. Moreover, mixed surfactant systems, such as cationic-anionic blends, achieved IFT values in the range of 7–10 mN/m at comparable concentrations and temperatures^18^. In contrast, the G2 C12 dendrimer reached a lower IFT at a relatively moderate concentration (2 g/L), suggesting enhanced interfacial activity. This improved performance can be attributed to the dendrimer’s amphiphilic structure, high surface functionality, and its ability to form a robust and densely packed interfacial layer^34–36^. These comparisons underscore the dendrimer’s superior ability to reduce capillary forces and improve oil mobilization, making it a competitive candidate for surfactant-based EOR under realistic reservoir conditions.

**Table 1:** Interfacial tension (IFT) values of cationic PAMAM dendrimer G2-C12 at different concentrations, including standard deviation (± SD).

Surfactant concentrations, ppm	IFT, mN/m	±SD (mN/m)
0 ppm (oil and DW)	30	± 0.00
0 ppm(oil and FW)	27	± 0.00
1 х 10^3^ ppm	10	± 0.32
1.5 х 10^3^ ppm	7	± 0.28
2 х 10^3^ ppm	5	± 0.24
2.5 х 10^3^ ppm	3	± 0.00
3 х 10^3^ ppm	2	± 0.37
3.5 х 10^3^ ppm	2	± 0.33

To further evaluate the performance of G2-C12 under varying reservoir conditions, the effect of brine salinity on interfacial tension (IFT) was studied. Surfactant solutions were prepared using synthetic formation waters with salinities of 10,000 ppm, 50,000 ppm, and 100,000 ppm. The results showed that IFT values decreased with lower salinity, indicating improved surfactant efficiency. At 2000 ppm surfactant concentration, the IFT decreased to approximately 3.2 mN/m in 10,000 ppm brine, 4.1 mN/m in 50,000 ppm brine, and 5.0 mN/m in 100,000 ppm brine. These findings confirm that higher salinity negatively impacts surfactant performance due to electrostatic screening and reduced molecular adsorption at the oil–water interface^37,38^. Results are summarized in Table 2.

The table presents the interfacial tension (IFT) values measured between crude oil and aqueous solutions of the G2-C12 surfactant at varying salinity levels ranging from 0 to 100,000 ppm. Additional measurements were performed at low salinity levels (500 and 1000 ppm) to determine the optimal salinity range for surfactant efficiency. Each value represents the average of three replicates, with the corresponding standard deviation (± SD) shown to indicate measurement reliability. The results reveal a minimum IFT near 2000 ppm, after which IFT increases with rising salinity.

**Table 2:** Effect of salinity on the interfacial tension (IFT) between crude oil and the G2-C12 dendrimer surfactant solution at 70 °C.

Salinity (ppm)	IFT (mN/m)	±SD (mN/m)
0	5.2	0.3
500	4.8	0.2
1000	4.5	0.2
2000	4.2	0.1
5000	4.7	0.2
10,000	5.1	0.3
25,000	6.2	0.3
50,000	7.3	0.4
100,000	9.0	0.4

### Wettability alteration

Wettability is a key parameter influencing oil recovery, as it governs the capillary forces and fluid distribution within the porous rock. Altering the rock surface from oil-wet to water-wet enhances the displacement efficiency of trapped oil, particularly in sandstone reservoirs. In this study, contact angle measurements were conducted on sandstone core surfaces before and after treatment with the G2-C12 dendrimer surfactant to evaluate wettability alteration.

Prior to surfactant injection, the sandstone cores exhibited strongly oil-wet characteristics, with contact angles exceeding 130°, indicating a strong affinity of the rock surface toward the oil phase. This behavior is typical in reservoirs where asphaltenes and other polar crude oil components adsorb onto the mineral surface, particularly on siliceous substrates such as quartz.

**Fig. 3:**
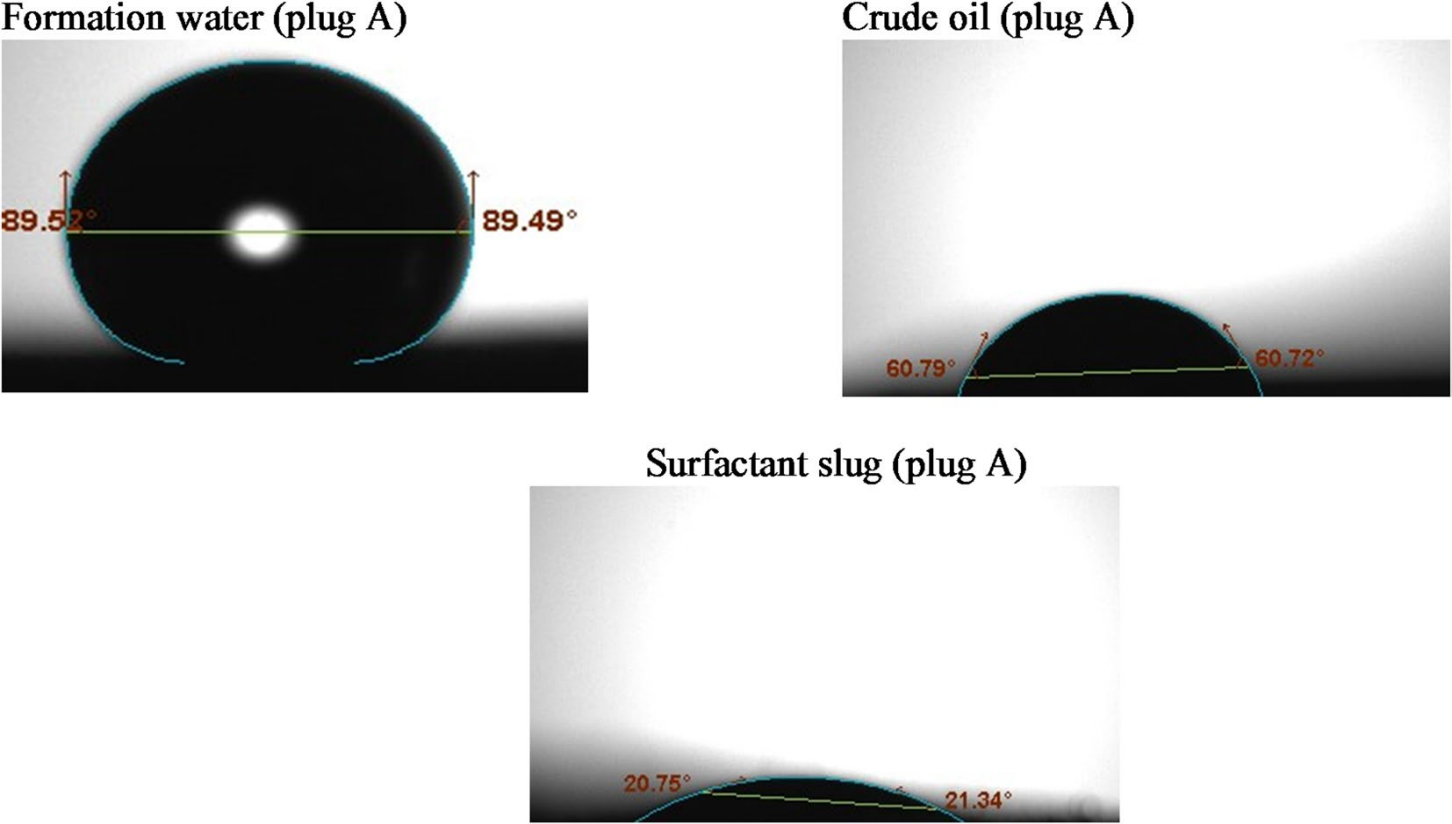
Contact angle measurements of the tested liquids on sandstone sample #A.

**Fig. 4:**
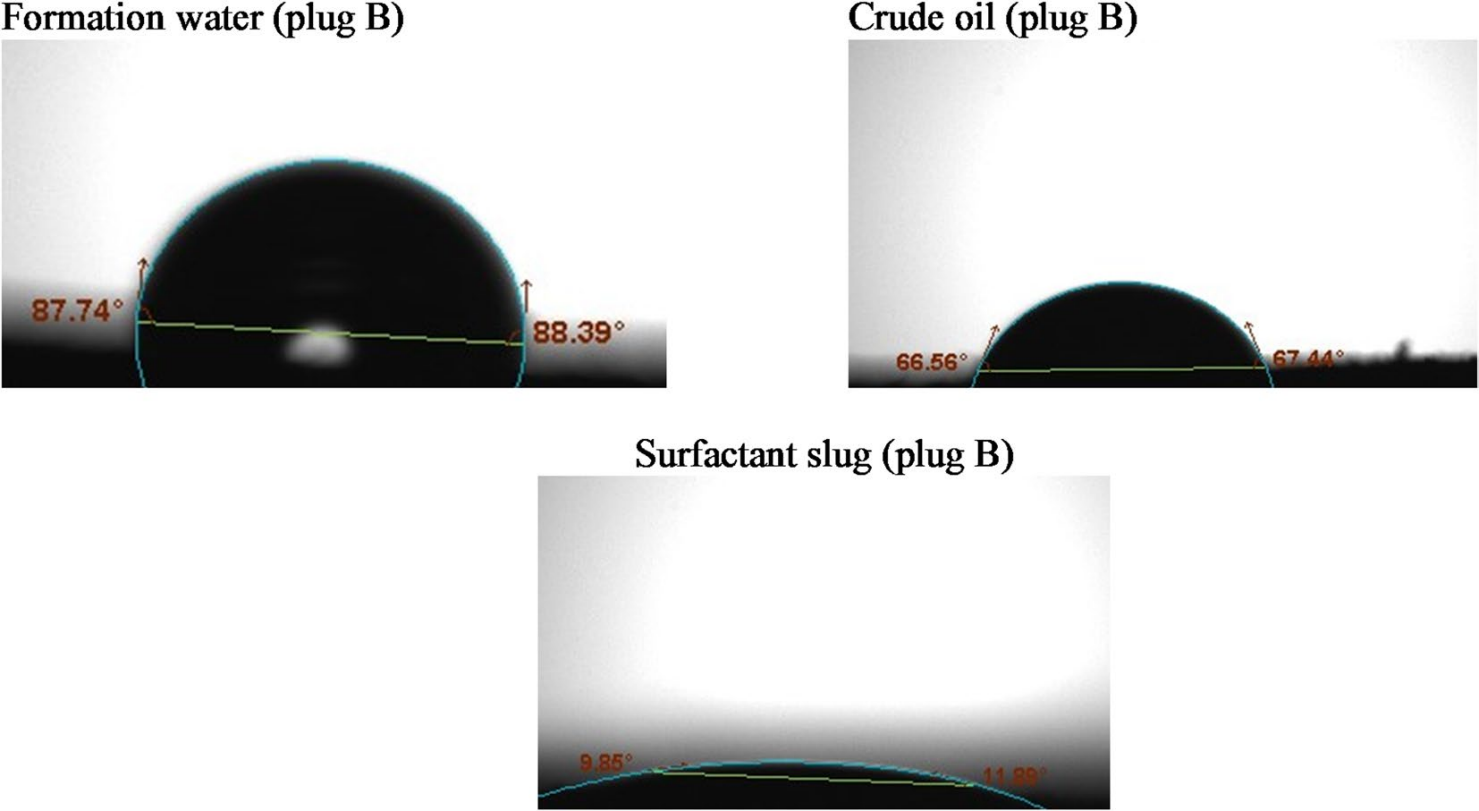
Contact angle measurements of the tested liquids on sandstone sample # B.

**Fig. 5:**
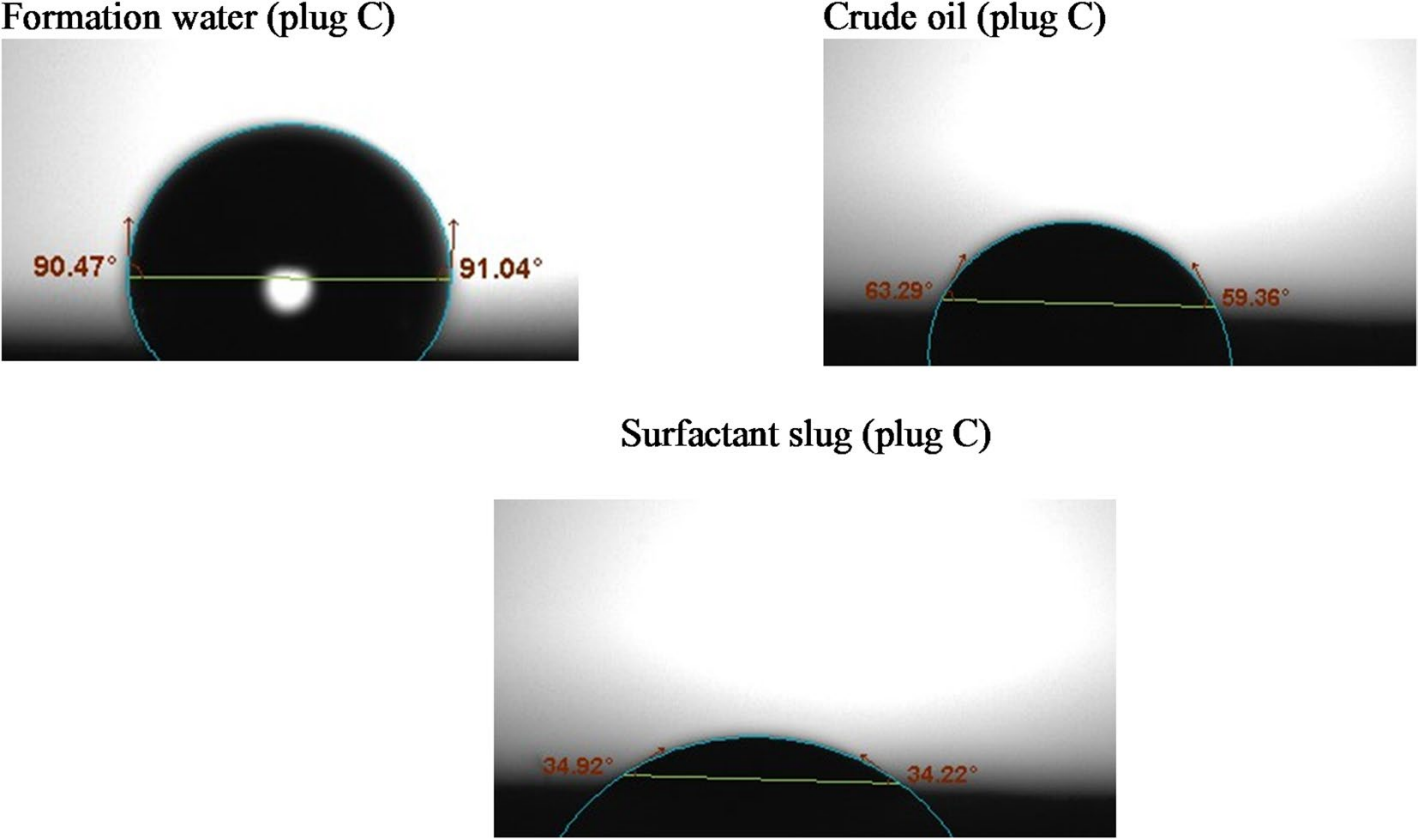
Contact angle measurements of the tested liquids on sandstone sample # C.

Following the injection of G2-C12 surfactant solution (2000 ppm), the contact angles were significantly reduced, ranging from 10.87° to 34.57° across the tested samples (A, B, and C), as shown in Figs. 3, 4 and 5. The detailed values are summarized in Table 3. This pronounced reduction indicates a clear shift from oil-wet to strongly water-wet conditions. The cationic nature of the dendrimer allows it to adsorb effectively onto negatively charged sandstone surfaces, displacing the adsorbed oil molecules and modifying the surface energy. The quaternary ammonium groups at the periphery of the dendrimer further enhance electrostatic attraction and interfacial disruption.

Although no XRD analysis was directly conducted in this study, previous geological reports on the Bahariya Formation sandstone indicate that it is predominantly composed of quartz, with minor amounts of feldspar and clay minerals. This quartz-rich nature of the rock is known to promote oil-wet behavior due to strong interactions with polar components in crude oil. The observed wettability alteration following treatment with the cationic G2-C12 dendrimer is consistent with the surfactant’s known affinity for siliceous surfaces, facilitating the transition toward water-wet conditions.

**Table 3:** Contact angle values of different fluids with each tested rock sample.

Tested sample	Fm. water			Crude oil			Surfactant slug		
	L°	*R*°	Av°	L°	*R*°	Av°	L°	*R*°	Av°
A	89.5	89.49	89.50	60.79	60.72	60.75	20.75	21.34	21.04
B	87.74	88.39	88.07	66.56	67.44	67	9.85	11.89	10.87
C	90.47	91.04	90.76	63.29	59.36	61.32	34.92	34.22	34.57

### Core flooding performance

To assess the practical efficiency of the G2-C12 dendrimer surfactant in enhanced oil recovery, core flooding experiments were conducted using actual sandstone cores under reservoir-representative conditions (70 °C and 100,000 ppm salinity). The experimental design included initial waterflooding with formation brine to establish residual oil saturation (Sor), followed by surfactant flooding at the optimal concentration (2000 ppm).

**Fig. 6:**
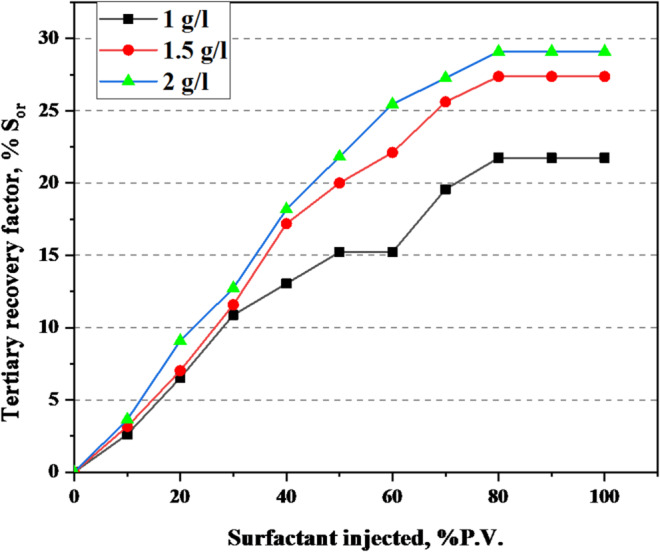
Oil recovery percentage (%) S_or_ by different surfactant concentrations.

**Fig. 7:**
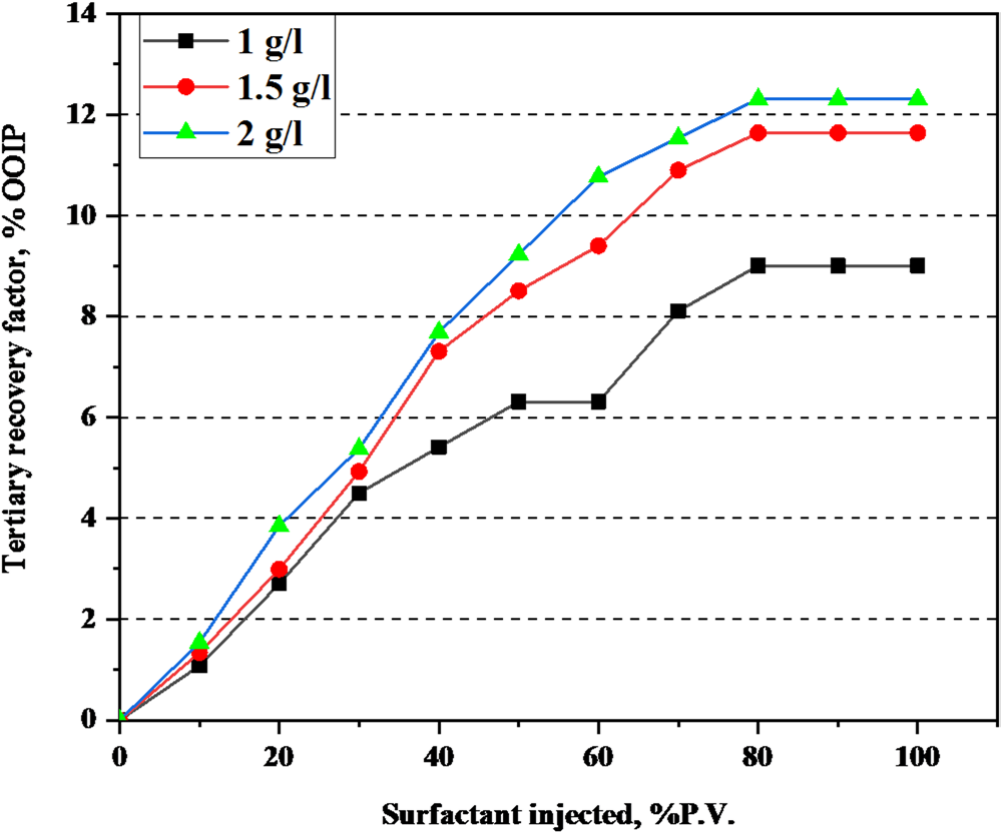
Oil recovery percentage (%) OOIP by different surfactant concentrations.

The incremental oil recovery achieved by surfactant flooding was substantial, reaching up to 29.09% of the original residual oil. The variation in oil recovery with surfactant concentration is illustrated in Figs. 6 and 7, showing recovery trends in terms of residual oil saturation (Sor) and original oil in place (OOIP), respectively. This recovery is significantly higher than values typically reported for conventional surfactants under similar high-salinity conditions. The enhanced performance is attributed to the dual effect of the G2-C12 dendrimer: (i) its strong interfacial tension reduction, facilitating capillary-driven oil mobilization, and (ii) its ability to alter rock wettability, promoting spontaneous imbibition and improving sweep efficiency.

The results observed in core samples A, B, and C were consistent, confirming the reproducibility and robustness of the dendrimer surfactant under reservoir-like conditions. The petrophysical properties of the plugs and flooding conditions are presented in Table 4, while the density and viscosity values of the tested liquids are listed in Table 5. Post-flooding observations showed improved oil phase continuity and reduced pressure differentials, indicating more efficient flow pathways and displacement dynamics.

Collectively, the findings from the IFT measurements, salinity optimization, wettability alteration, and core flooding demonstrate that the G2-C12 dendrimer surfactant operates through multiple synergistic mechanisms that enhance oil recovery. The cumulative oil recovery profile for plug A at 1 g/L surfactant concentration is shown in Table 6, while the corresponding results for plug B (1.5 g/L) and plug C (2 g/L) are presented in Tables 7 and 8, respectively. This validates its potential as a high-performance chemical EOR agent, particularly suited for harsh reservoir environments where conventional surfactants underperform^39^.

**Table 4:** The petrophysical properties of the tested plugs and flooding conditions.

Parameters	Value		
Plugs properties at reservoir conditions			
Plug ID	A	B	C
Length, cm	4.334	4.538	4.735
Diameter, cm	3.827	3.824	3.828
Area, cm^2^	11.50	11.48	11.51
Bulk volume (B.V.), cc	49.85	52.12	53.49
Pore volume (P.V.), cc	10.63	11.2	10.78
Grain volume (G.V.), cc	39.22	40.92	42.72
Porosity (%)	21.3	21.5	20.14
Volume of oil injected, cc	5.55	6.7	6.5
S_wi_, cc	5.08	4.50	4.28
S_or_, cc	2.3	2.85	2.75
Initial water saturation (S_wi_), % P.V.	47.79	40.18	39.70
Initial oil saturation (S_oi_), % P.V.	52.21	59.82	60.30
Residual oil saturation (S_or_), % P.V.	21.63	25.44	25.51
Residual oil saturation (S_or_), % OOIP	41.44	42.53	42.31
Flooding conditions			
Flooding temperature, °C	70		
Confining pressure, psi	4000		
Brine salinity, ppm	100,000		

**Table 5:** Density, viscosity, and API values of the tested liquids.

Liquid	Conditions	Density, gm/cc	Dynamic viscosity, Cp	API
Fm. water	Ambient	1.138	1.55	–
	70 °C	1.108	0.88	–
Crude oil	Ambient	0.921	50.8	29.74
	70 °C	0.884	9.39	32.51

**Table 6:** Cumulative oil recovery as a function of pore volume injected in the case of (A) (1 g/l).

Slug injected	Tertiary oil recovery	Tertiary oil recovery
% P.V.	%, s_or_	% OOIP
0	0	0
10	2.61	1.08
20	6.52	2.70
30	10.87	4.50
40	13.04	5.41
50	15.22	6.31
60	15.22	6.31
70	19.57	8.11
80	21.74	9.01
90	21.74	9.01
100	21.74	9.01

**Table 7:** Cumulative oil recovery as a function of pore volume injected in the case of (B) (1.5 g/l).

Slug injected	Tertiary oil recovery	Tertiary oil recovery
% P.V.	%, s_or_	% OOIP
0	0	0
10	3.16	1.34
20	7.02	2.99
30	11.58	4.93
40	17.19	7.31
50	20.00	8.51
60	22.11	9.40
70	25.61	10.90
80	27.37	11.64
90	27.37	11.64
100	27.37	11.64

**Table 8:** Cumulative oil recovery as a function of pore volume injected in the case of (D) (2 g/l).

Slug injected	Tertiary oil recovery	Tertiary oil recovery
% P.V.	%, s_or_	% OOIP
0	0	0
10	3.64	1.54
20	9.09	3.85
30	12.73	5.38
40	18.18	7.69
50	21.82	9.23
60	25.45	10.77
70	27.27	11.54
80	29.09	12.31
90	29.09	12.31
100	29.09	12.31

**Fig. 8:**
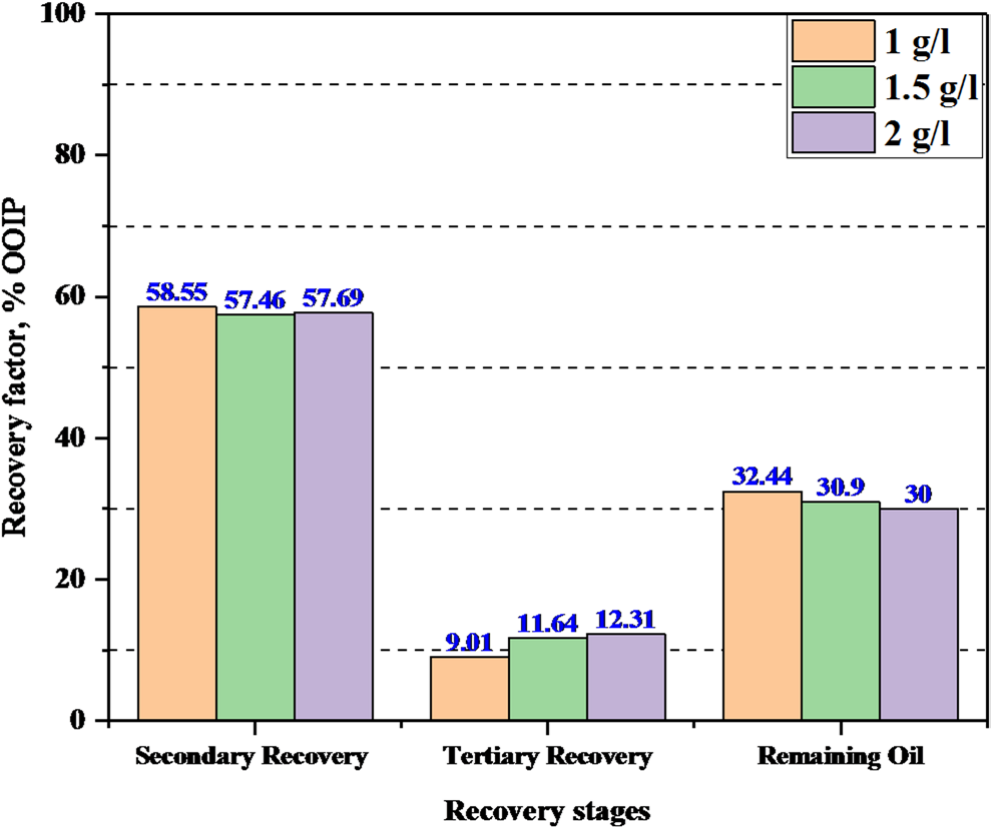
Histogram displaying recovery stages and the remaining oil using different surfactant concentrations.

The recovery factor is determined by summing the oil recovered during each stage of the process, including secondary and tertiary displacement. The calculations in Table 9 indicated positive economics for enhanced oil recovery through the novel surfactant where it can withstand severe reservoir conditions and enhance the recovery factor (Fig. 8).

Compared with oil displacement agents used in previous studies, the synthesized G2-C12 dendrimer surfactant demonstrated several key advantages over conventional oil displacement agents reported in previous studies. One of the most notable benefits is its high oil recovery efficiency under harsh reservoir conditions, including high temperature (70 °C) and high salinity (100,000 ppm), where many traditional surfactants tend to degrade or lose effectiveness. In this study, G2-C12 achieved a maximum oil recovery of 29.09% of residual oil saturation (S_or_) at 2000 ppm, a performance that is higher or at least comparable to conventional surfactants used at similar or even higher concentrations. For example, sodium lauryl sulfate and sodium dodecyl sulfate typically yield recovery improvements in the range of 4–16% under comparable conditions^15,33^. Moreover, the amphiphilic and nanoscale structure of G2-C12 enables it to penetrate deeply into porous media, effectively reduce IFT, and strongly alter rock wettability. Unlike conventional surfactants that often suffer from high adsorption losses on rock surfaces, dendrimer-based systems benefit from structural uniformity and reduced adsorption, enhancing their economic and operational viability. These unique properties make G2-C12 a robust and efficient EOR agent, particularly suitable for application in complex reservoir environments where conventional agents fail to maintain stability or performance^40^.

**Table 9:** Secondary and tertiary recovery factors during the flooding process using different concentrations of the new surfactant agent.

Parameter			Plug (A) (1 g/l)						Plug (B) (1.5 g/l)			Plug (C) (2 g/l)		
V_o_ injected, cc			5.55						6.7			6.5		
V_o_ recovered by PM and SM, cc			3.25						3.85			3.75		
V_o_ remain, cc			2.3						2.85			2.75		
Recovered oil by TM, cc			RF_PM+SM_ (%OOIP)			RF_TM_ (%OOIP)			RF_Total_ (%OOIP)			Remaining oil (%OOIP)		
**A**	**B**	**C**	**A**	**B**	**C**	**A**	**B**	**C**	**A**	**B**	**C**	**A**	**B**	**C**
0.5	0.78	0.8	58.55	57.46	57.69	9.01	11.64	12.31	67.56	69.10	70.00	32.44	30.90	30.00

For comparison, core flooding experiments were also conducted using a conventional anionic surfactant, sodium lauryl sulfate (SLS), under the same reservoir-representative conditions (70 °C, 100,000 ppm brine, identical sandstone cores, and similar injection protocols). The SLS solution was prepared at its optimum concentration of 2 g/L, determined from preliminary IFT measurements. As shown in Table 10, SLS achieved an incremental oil recovery of 15.84% of the residual oil saturation (Sor), which is significantly lower than the 29.09% obtained with the synthesized G2-C12 dendrimer surfactant at the same concentration. The lower performance of SLS is attributed to its reduced stability under high salinity, leading to partial precipitation and lower efficiency in wettability alteration. This direct, side-by-side comparison confirms that the G2-C12 dendrimer offers superior oil displacement capability under harsh reservoir conditions.

**Table 10:** Incremental oil recovery comparison between conventional surfactant (SLS) and G2-C12 dendrimer under identical reservoir conditions.

Surfactant type	Concentration (g/L)	Incremental oil recovery (% Sor)	Incremental oil recovery (% OOIP)
Sodium lauryl sulfate (SLS)	2.0	15.84	6.70
G2-C12 dendrimer	2.0	29.09	12.31

**Fig. 9:**
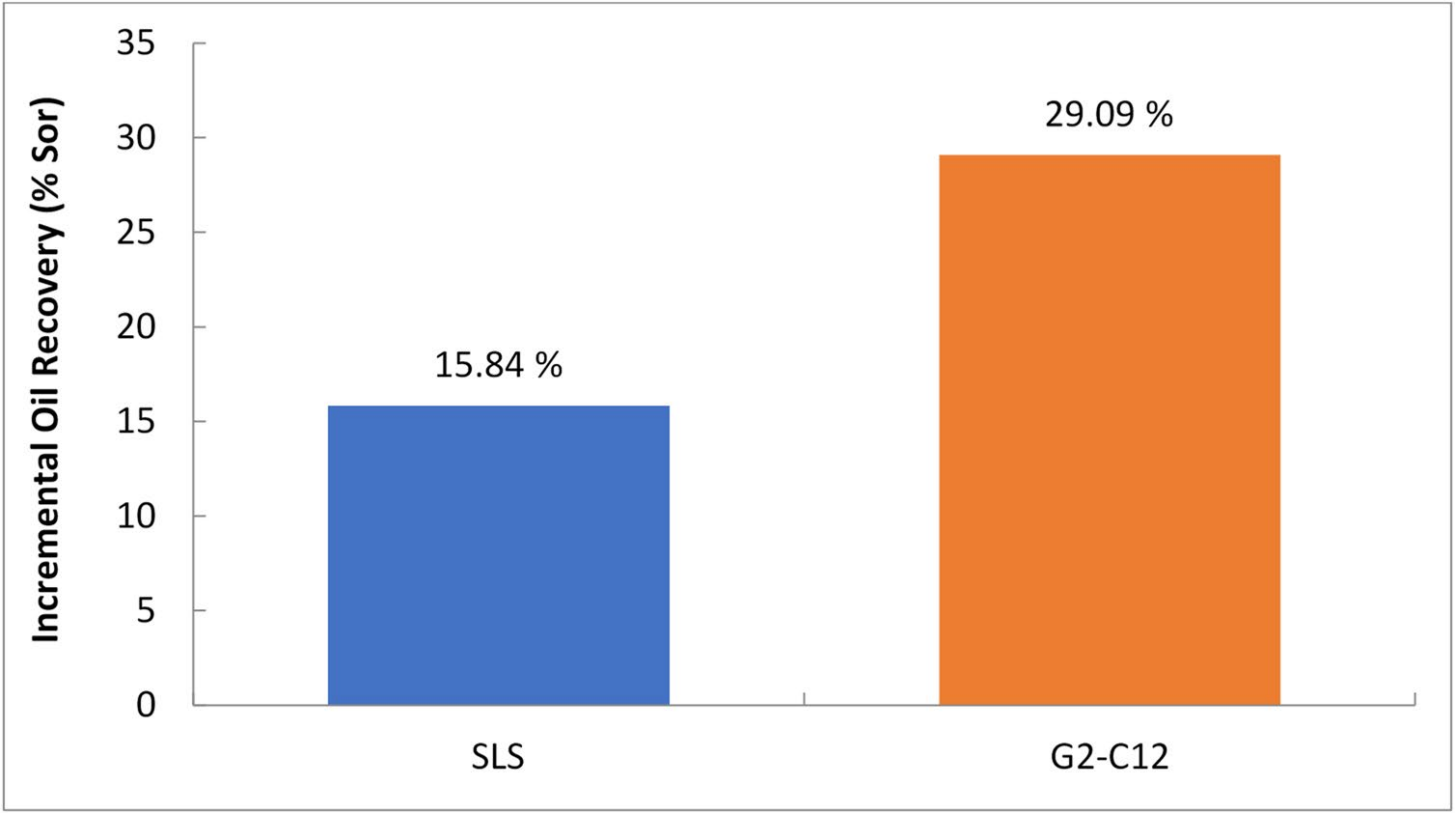
Comparison of incremental oil recovery for SLS and G2-C12.

The comparison between the incremental oil recovery of sodium lauryl sulfate (SLS) and G2-C12 dendrimer under identical reservoir conditions is illustrated in Fig. 9. The G2-C12 dendrimer achieved almost double the oil recovery efficiency of SLS, highlighting its superior performance in high-salinity, high-temperature environments.

### Challenges

Despite its promise, implementing PAMAM dendrimer surfactants in EOR faces several challenges:

- **Cost and Scalability**: Manufacturing dendrimers at industrial scales can be cost-intensive.
- **Reservoir Heterogeneity**: Variations in permeability and rock properties may affect uniform surfactant distribution.
- **Environmental Concerns**: Ensuring the surfactant’s biodegradability and minimal ecological impact remains a priority.
- **Compatibility**: Interaction with reservoir fluids and minerals could influence performance.
- Additionally, the rheological behavior of the G2-C12 surfactant solution was not investigated in this study. Given the highly branched and amphiphilic structure of the dendrimer, it is expected that the surfactant may exhibit non-Newtonian flow behavior under reservoir conditions. Such properties could influence injectivity, flow mobility, and sweep efficiency. Therefore, future work will involve a comprehensive rheological analysis to evaluate shear-dependent viscosity and ensure the practical applicability of the surfactant under high-temperature and high-salinity conditions.
- Moreover, the current study did not investigate the emulsification behavior of the G2-C12 surfactant, which is known to play a key role in enhanced oil recovery by stabilizing dispersed oil or water droplets and aiding mobilization. Future work will incorporate a detailed emulsification study, including emulsion type determination, droplet size distribution, and stability assessment under reservoir conditions to better understand the role of emulsification in the observed oil recovery.

Addressing these challenges requires further research and optimization to fully realize the potential of PAMAM dendrimers in EOR applications.

## Conclusion

This study demonstrated the effectiveness of a quaternized cationic PAMAM dendrimer (G2-C12) as a novel surfactant for enhanced oil recovery (EOR) in sandstone reservoirs under high-salinity and high-temperature conditions. Through a series of interfacial tension (IFT) measurements, salinity sensitivity evaluations, wettability alteration tests, and core flooding experiments, the G2-C12 surfactant exhibited superior performance compared to conventional systems. The surfactant significantly reduced IFT from 27 to 5 mN/m, with a critical micelle concentration (CMC) identified at 2000 ppm. Additional salinity tests confirmed optimal performance near this value, highlighting the dendrimer’s tolerance to brine strength. Contact angle measurements confirmed a strong wettability alteration from oil-wet to water-wet, supported by the known interaction between cationic surfactants and siliceous minerals. Core flooding tests yielded an impressive 29.09% additional oil recovery from residual oil, confirming the practical efficiency of the surfactant in reservoir-like conditions. The combined effects of strong interfacial activity and wettability reversal underline the dendrimer’s dual-action mechanism. Given its molecular uniformity, structural versatility, and functional performance, G2-C12 represents a promising candidate for field-scale application in harsh reservoir environments where conventional surfactants are ineffective. Future work may focus on its stability over prolonged injection periods, scale-up feasibility, and synergistic behavior when blended with other EOR agents.

## Data Availability

The datasets used and/or analyzed during the current study are available from the corresponding author on reasonable request.

## Abbreviations

PAMAM: Cationic hyperbranched polyamidoamine
EOR: Enhanced oil recovery
ITF: Interfacial tension
S_or_: Residual oil saturation
B_V_: Bulk volume
GV: Grain volume
PV: Pore volume
Ø: Porosity
MnCl_2_: Magnesium chloride
FTIR: Fourier-transform infrared spectroscopy
^1^HNMR: Proton nuclear magnetic resonance
TGA: Thermal gravimetric analysis
DLS: Dynamic light scattering
cSt: Centistokes (unit of kinematic viscosity)
cP: Centipoise (unit of dynamic viscosity)
SG: Specific gravity
API: American petroleum institute
psi: Pound per square inch (unit of pressure)
OOIP: Original oil in place
S_wi_: Initial water saturation
NaCl: Sodium chloride
CaCl: Calcium chloride
Dw: Deionized water
Fw: Formation water
S_oi_: Initial oil saturation
PM: Primary method
SM: Secondary method
TM: Tertiary method
RF_PM+SM_: Recovery factor obtained by primary and secondary methods
RF_TM_: Recovery factor obtained by tertiary method
RF_Total_: Total recovery factor
